# Supplementary material for: Functional histology of the skin in the subterranean African giant mole-rat: thermal windows are determined solely by pelage characteristics
Source: PeerJ. 2020 Apr 8;8:e8883. doi: 10.7717/peerj.8883 (PMC7150539; doi:10.7717/peerj.8883)
Supplement: Supplemental Information 1 — Proportion (in % of the whole sample width) of each fat layer thickness category in the skin samples of each specimen. L, left body side; R, right body side; SL, Sampling locations: 1 – anterior, 3 – middle, 5 – posterior, see Fig. 1. [file peerj-08-8883-s001.docx]

| Animal ID | Body part | | SL | Fat tissue thickness categories (µm) | | | | | | |
| --- | --- | --- | --- | --- | --- | --- | --- | --- | --- | --- |
|  |  | |  | 0 | 1-50 | 50-100 | 100-150 | 150-200 | 200-250 | >250 |
| 2296 | Dorsum L | | 1 | 13.4 | 37.1 | 36.4 | 13.3 | 0.0 | 0.0 | 0.0 |
|  |  | | 3 | 3.7 | 5.5 | 22.9 | 30.6 | 26.3 | 9.6 | 1.6 |
|  |  | | 5 | 0.0 | 0.0 | 5.2 | 20.1 | 23.1 | 20.9 | 30.8 |
|  | Dorsum R | | 1 | 3.6 | 55.9 | 33.6 | 7.0 | 0.0 | 0.0 | 0.0 |
|  |  | | 3 | 0.0 | 13.2 | 36.5 | 46.4 | 4.0 | 0.0 | 0.0 |
|  |  | | 5 | 0.0 | 0.0 | 0.0 | 19.7 | 32.1 | 29.0 | 19.3 |
|  | Venter L | | 1 | 0.0 | 2.7 | 14.9 | 15.7 | 41.1 | 20.3 | 5.4 |
|  |  | | 3 | 0.0 | 12.7 | 16.2 | 29.0 | 31.6 | 10.5 | 0.0 |
|  |  | | 5 | 0.0 | 0.0 | 0.0 | 13.2 | 24.4 | 29.4 | 33.0 |
|  | Venter R | | 1 | 15.9 | 39.6 | 44.5 | 0.0 | 0.0 | 0.0 | 0.0 |
|  |  | | 3 | 19.2 | 27.9 | 19.8 | 15.6 | 12.2 | 5.3 | 0.0 |
|  |  | | 5 | 0.0 | 9.8 | 9.2 | 27.9 | 27.5 | 12.7 | 12.9 |
| 7940 | Dorsum L | | 1 | 0.0 | 9.2 | 7.5 | 15.8 | 21.4 | 18.0 | 28.1 |
|  |  | | 3 | 0.0 | 0.0 | 2.8 | 8.8 | 35.9 | 33.1 | 19.5 |
|  |  | | 5 | 0.0 | 5.1 | 17.3 | 41.2 | 33.6 | 3.0 | 0.0 |
|  | Dorsum R | | 1 | 0.5 | 7.1 | 24.7 | 21.4 | 21.2 | 13.6 | 11.5 |
|  |  | | 3 | 0.0 | 9.3 | 27.0 | 30.1 | 26.1 | 7.5 | 0.0 |
|  |  | | 5 | 1.2 | 9.7 | 27.5 | 36.8 | 13.1 | 11.7 | 0.0 |
|  | Venter L | | 1 | 26.8 | 28.2 | 22.2 | 22.9 | 0.0 | 0.0 | 0.0 |
|  |  | | 3 | 2.1 | 8.2 | 14.9 | 34.4 | 23.4 | 15.3 | 1.8 |
|  |  | | 5 | 5.8 | 3.3 | 18.7 | 12.3 | 15.9 | 28.0 | 16.1 |
|  | Venter R | | 1 | 0.0 | 16.6 | 39.2 | 24.5 | 8.6 | 11.1 | 0.0 |
|  |  | | 3 | 10.4 | 14.8 | 18.6 | 33.5 | 14.0 | 4.3 | 4.5 |
|  |  | | 5 | 16.1 | 18.3 | 28.7 | 36.9 | 0.0 | 0.0 | 0.0 |
| 8280 | Dorsum L | | 1 | 4,3 | 13,5 | 27,3 | 48,7 | 6,2 | 0,0 | 0,0 |
|  |  | | 3 | 0,0 | 41,9 | 31,4 | 20,8 | 5,9 | 0,0 | 0,0 |
|  |  | | 5 | 4,1 | 42,2 | 32,9 | 20,8 | 0,0 | 0,0 | 0,0 |
|  | Dorsum R | | 1 | 5,2 | 27,3 | 18,6 | 29,7 | 19,2 | 0,0 | 0,0 |
|  |  | | 3 | 3,8 | 22,8 | 38,8 | 21,3 | 13,3 | 0,0 | 0,0 |
|  |  | | 5 | 4,0 | 27,2 | 39,0 | 23,2 | 6,6 | 0,0 | 0,0 |
|  | Venter L | | 1 | 5,3 | 47,0 | 33,1 | 14,6 | 0,0 | 0,0 | 0,0 |
|  |  | | 3 | 4,7 | 41,9 | 39,7 | 13,7 | 0,0 | 0,0 | 0,0 |
|  |  | | 5 | 0,0 | 0,0 | 25,4 | 47,7 | 26,9 | 0,0 | 0,0 |
|  | Venter R | | 1 | 6,6 | 30,6 | 41,5 | 9,2 | 6,1 | 6,0 | 0,0 |
|  | |  | 3 | 0,0 | 10,6 | 75,5 | 13,9 | 0,0 | 0,0 | 0,0 |
|  | |  | 5 | 4,5 | 37,7 | 33,5 | 24,3 | 0,0 | 0,0 | 0,0 |
| 9330 | Dorsum L | | 1 | 0,0 | 0,0 | 2,4 | 14,1 | 34,1 | 28,3 | 21,1 |
|  |  | | 3 | 0,0 | 0,0 | 0,0 | 23,4 | 27,6 | 27,3 | 21,7 |
|  |  | | 5 | 0,0 | 0,0 | 6,3 | 27,4 | 24,0 | 27,4 | 14,9 |
|  | Dorsum R | | 1 | 0,0 | 9,9 | 8,6 | 20,5 | 34,5 | 14,6 | 11,9 |
|  |  | | 3 | 0,0 | 15,0 | 15,8 | 21,4 | 19,0 | 22,6 | 6,2 |
|  |  | | 5 | 0,0 | 0,0 | 7,5 | 20,1 | 47,4 | 9,5 | 15,5 |
|  | Venter L | | 1 | 0,0 | 14,5 | 13,7 | 48,5 | 6,5 | 9,9 | 6,9 |
|  |  | | 3 | 2,8 | 18,2 | 61,5 | 9,1 | 8,4 | 0,0 | 0,0 |
|  |  | | 5 | 0,0 | 19,3 | 37,0 | 27,2 | 16,5 | 0,0 | 0,0 |
|  | Venter R | | 1 | 0,0 | 41,2 | 39,8 | 19,0 | 0,0 | 0,0 | 0,0 |
|  | |  | 3 | 4,8 | 33,4 | 32,0 | 19,7 | 10,1 | 0,0 | 0,0 |
|  | |  | 5 | 0,0 | 9,7 | 31,8 | 45,3 | 13,2 | 0,0 | 0,0 |
| 9653 | Dorsum L | | 1 | 0.0 | 5.8 | 8.8 | 9.0 | 26.0 | 20.6 | 29.9 |
|  |  | | 3 | 0.0 | 6.4 | 8.1 | 29.7 | 36.6 | 11.5 | 7.9 |
|  |  | | 5 | 0.0 | 14.0 | 49.4 | 36.6 | 0.0 | 0.0 | 0.0 |
|  | Dorsum R | | 1 | 0.0 | 4.1 | 4.4 | 14.7 | 32.1 | 24.8 | 19.9 |
|  |  | | 3 | 0.0 | 5.0 | 32.5 | 43.0 | 11.1 | 8.3 | 0.0 |
|  |  | | 5 | 2.5 | 20.4 | 48.3 | 26.0 | 2.8 | 0.0 | 0.0 |
|  | Venter L | | 1 | 7.1 | 14.2 | 30.8 | 47.9 | 0.0 | 0.0 | 0.0 |
|  |  | | 3 | 1.8 | 7.6 | 34.1 | 31.0 | 25.6 | 0.0 | 0.0 |
|  |  | | 5 | 0.0 | 18.4 | 24.9 | 37.3 | 19.5 | 0.0 | 0.0 |
|  | Venter R | | 1 | 0.0 | 5.3 | 11.4 | 28.4 | 44.6 | 10.3 | 0.0 |
|  | |  | 3 | 0.0 | 3.2 | 11.0 | 14.6 | 23.6 | 31.8 | 15.8 |
|  | |  | 5 | 0.0 | 7.0 | 30.0 | 55.5 | 7.6 | 0.0 | 0.0 |
