# Supplementary material for: Functional histology of the skin in the subterranean African giant mole-rat: thermal windows are determined solely by pelage characteristics
Source: PeerJ. 2020 Apr 8;8:e8883. doi: 10.7717/peerj.8883 (PMC7150539; doi:10.7717/peerj.8883)
Supplement: Supplemental Information 2 — Sampling location is marked from anterior (1) to posterior (5) part see Fig. 1. [file peerj-08-8883-s002.docx]

| Animal ID | Body part | Sampling location | Left side vessel percentage | Right side vessel percentage |
| --- | --- | --- | --- | --- |
| 8280 | Dorsum | 1 | 21.9 | 19.3 |
|  |  | 2 | 24.7 | 22 |
|  |  | 3 | 22 | 21.1 |
|  |  | 4 | 20.1 | 19.8 |
|  |  | 5 | 22 | 20 |
|  | Ventrer | 1 | 28.1 | 34.8 |
|  |  | 2 | 21.8 | 28.5 |
|  |  | 3 | 24.3 | 21.9 |
|  |  | 4 | 36.4 | 27.5 |
|  |  | 5 | 25.2 | 28.7 |
| 9330 | Dorsum | 1 | 20.1 | 25 |
|  |  | 2 | 22.8 | 22.4 |
|  |  | 3 | 24.5 | 20.3 |
|  |  | 4 | 25 | 25.6 |
|  |  | 5 | 23.8 | 21.3 |
|  | Venter | 1 | 27.3 | 26.3 |
|  |  | 2 | 29.2 | 24.4 |
|  |  | 3 | 29 | 24.4 |
|  |  | 4 | 24.8 | 23.3 |
|  |  | 5 | 24.1 | 23.8 |
| 9653 | Dorsum | 1 | 23.5 | 21.4 |
|  |  | 2 | 21.4 | 25.6 |
|  |  | 3 | 26.8 | 28.7 |
|  |  | 4 | 23.8 | 20.3 |
|  |  | 5 | 20.6 | 23.6 |
|  | Venter | 1 | 25.6 | 26.3 |
|  |  | 2 | 25.6 | 29.2 |
|  |  | 3 | 26 | 23.6 |
|  |  | 4 | 27.5 | 27.9 |
|  |  | 5 | 24.2 | 25 |
